# PROPOSAL OF A REVISIONAL SURGERY TO TREAT NON-INSULINOMA HYPERINSULINEMIC HYPOGLICEMIA POSTGASTRIC BYPASS

**DOI:** 10.1590/S0102-6720201500040015

**Published:** 2015

**Authors:** José SAMPAIO-NETO, Alcides José BRANCO-FILHO, Luis Sérgio NASSIF, André Thá NASSIF, Flávia David João De MASI, Daniele Rezende XIMENEZ

**Affiliations:** Bariatric Surgery Service, Santa Casa de Misericórdia Hospital, Curitiba, PR, Brazil

**Keywords:** Bariatric surgery, Hyperinsulinemic hypoglycemia, Gastric bypass, Y-de-Roux, Gastroplasty

## Abstract

***Background*:**

Hyperinsulinemic hypoglicemia with severe neuroglycopenic symptoms has been identified as a late and rare complication in patients submitted to Roux-en-Y gastric bypass. However, the potential gravity of its manifestations requires effective treatment of this condition. The absence of treatment makes it necessary to develop more effective clinical or surgical methods.

***Aim*:**

To present one surgical option to revisional surgery in the treatment of hyperinsulinemic hypoglicemia

***Methods*:**

The procedure consists in reconstituting alimentary transit through the duodenum and proximal jejunum, while keeping the restrictive part of the gastric bypass. As an additional strategy to maintain weight loss, is realized gastric fundus resection, aiming to suppress ghrelin production more effectively.

***Results*:**

It was used in three patients with successful results in one year of follow-up.

***Conclusion*:**

The procedure to reconstruct the food transit through the duodenum and proximal jejunum, keeping the restrictive component of gastric bypass in the treatment of hyperinsulinemic hypoglycemia showed good initial results and validated its application in other cases with this indication.

## Abstract (pt)

***Racional*:**

Hipoglicemia hiperinsulinêmica com sintomas neuroglicopênicos severos tem sido identificada como complicação tardia e rara em pacientes submetidos à gastroplastia com bypass em Y-de-Roux. Porém, a gravidade potencial de suas manifestações exige tratamento definitivo desta condição. A falta de tratamento efetivo gera a necessidade de desenvolver métodos clínicos ou cirúrgicos mais eficazes.

**Objetivo:**

Apresentar proposta de operação revisional para o tratamento da síndrome de hipoglicemia hiperinsulinêmica.

O procedimento consiste em reconstituir o trânsito alimentar pelo duodeno e jejuno proximal, mantendo o componente restritivo do by-pass gástrico. Como estratégia adicional de manutenção ponderal, é realizada ressecção do fundo gástrico, visando intensificar a supressão da grelina.

**Resultado:**

O procedimento foi realizado em três pacientes com bom resultado em seguimento de um ano.

***Conclusão*:**

O procedimento de reconstituir o trânsito alimentar pelo duodeno e jejuno proximal, mantendo o componente restritivo do by-pass gástrico no tratamento da hipoglicemia hiperinsulinêmica apresentou bons resultados iniciais podendo validar sua indicação para outros casos.

## INTRODUCTION

To date, bariatric surgery has shown the best results in weight loss and maintenance of this loss in long follow-up^6^^,^ 11. Greater part of the population undergoing the surgical procedure also experience full or partial remission of comorbidities related to obesity, such as type 2 diabetes mellitus, hypertension and dislipidemia^3^^,^ 4 ^,^ 6.

The Roux-en-Y gastric bypass is the procedure performed worldwide, accounting for about 60% of all bariatric operations in 2008^5^. The main mechanisms related to improvement of obesity and its comorbidities triggered by gastric bypass are the effect on limiting caloric intake, duodenal and ileal bypass stimulation, leading to increased secretion incretinic factors such as GLP-1 and peptide-YY^2^^,^ 11. The latter has important effect on pancreatic release of insulin, an important factor in glycemic control 2 ^,^ 3

But recently, cases of hyperinsulinemic hypoglycemia have been reported, usually related to hyperplasia of pancreatic beta cells secondary to stimulation of ileal incretinic hormones. A syndrome called non-insulinoma pancreatogenous hyperinsulinemic hypoglycemia, syndrome - NIPHS includes neuroglycopenic symptoms such as cognitive impairment, behavioral changes, confusion, depression, sweating, weakness, dizziness, and, if severe hypoglycemia, generalized or focal seizures and coma, associated with documented hypoglycemia^9^. The estimated prevalence of this type of manifestation is probably underestimated, due to its similarity with clinical manifestations of dumping syndrome^3^.

Patients with such documented postprandial symptoms should be investigated in order to rule out other causes of hyperinsulinemic hypoglycemia, as the factitious (by administration of exogenous insulin or similar agents) and by insulinoma, being essential value the presence of normal or increased dosages of peptide-C and proinsulin in addition to the disposal by imaging of focal pancreatic lesions, to confirm NIPHS as the cause^1^^,^ 10 ^,^ 14 ^,^ 15.

Despite the affordable diagnosis, there is no consensus on the best treatment of the syndrome in the postoperative bariatric surgery, specifically gastric bypass. Initial therapy to be instituted, consisting of dietary and behavioral measures, has modest response, even when added the use of medications such as acarbose^8^. Several surgical proposals have been made in the literature, in which the pancreatic resections are usually employed. However, the high morbidity and recurrence rate of hypoglycemic events bring doubt on its real value^13^.

Treatments aimed at restoring the intestinal transit, as the reversal of prior bariatric surgery or revision to vertical gastrectomy showed good result in the resolution of episodes of reactive hypoglycemia, especially in cases where there was good response to preoperative nutrition by gastrostomy tactic that simulates the reconstitution effect of the transit through the duodenum and proximal jejunum. This is probably due to the release of anti-incretinic factors for these portions of the digestive tract when stimulated^7^^,^ 12.

Even with good response, complete reversal of gastric bypass in Roux-en-Y results in the undesired effect of returning to the original anatomy, which can lead to full regained the weight lost and returning the preoperative comorbidities. The review for vertical gastrectomy, while providing the benefits related to this technique is complex and high morbidity rate, mainly related to the occurrence of gastric fistula.

The objective of this paper is to present proposal for revisional surgery for the treatment of NIPHS through new surgical procedure, technically simpler than the existing alternatives, based on the pathophysiology of the condition and maintenance of weight loss.

## METHODS

The study was approved by the Ethics Committee on Research of the Hospital Santa Casa, Curitiba, PR, Brazil. Patients signed the free and informed consent form for the surgery.

The procedure consisted of laparoscopic access followed by release of supramesocolic adhesions. After identification of the bypassed stomach, it was held release of the greater curvature vessels with ultrasonic scalpel, followed by resection of the background and part of the gastric body with endoscopic stapler, and strengthening clamps line with manual suture.

**FIGURE 1:**
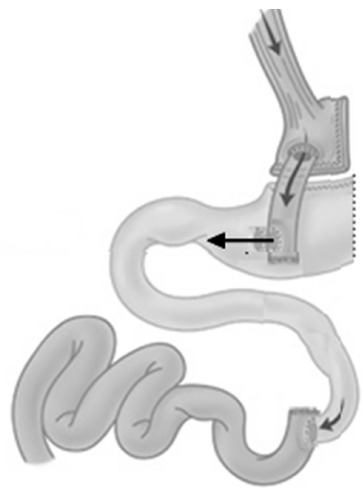
- Proposal for a revisional procedure for treating hyperinsulinemic hypoglycemia syndrome after bariatric surgery

Then was proceeded the section of food loop next to enteroenteric original anastomosis, proximal to it. The distal portion of feed loop was then anastomosed laterally with gastric antrum 4 cm from the pylorus (Figure 1). At the end, anastomosis was performed with methylene blue test; there was no cavity drainage.

**FIGURE 2:**
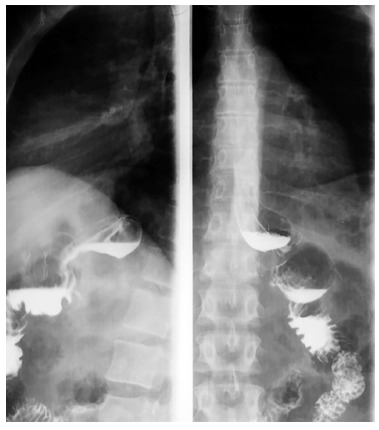
- Contrast radiography illustrating the result of the proposed revisional operation

Patients were fasted orally until the second day after surgery, when it was release restrict liquid diet and hospital discharge. It was conducted clinical and laboratory outpatient monitoring after 15 days, 45 days, three months and at each three months to the end of the first year. The postoperative radiologic appearance after oral intake contrast can be seen in Figure 2.

## RESULTS

The procedure was performed in three cases through laparoscopy in patients previously operated for 8, 9 and 11 years. They presented before the bariatric procedure comorbidities such as hypertension, fatty liver and dyslipidemia, with poor control and that were solved with the initial procedure. They were three women with significant weight loss after performing the first surgical procedure (Table 1).

**TABLE 1:** - Demographic data of patients who underwent the proposed revisional operation

**Age**	**Gender**	**Height**	**Weight/IM pre-operative**	**Weight/IM pre-reoperative**
32	F	1,50	91 / 40,4	69 / 30,6
43	F	1,75	112,7 / 36,8	93 / 30,1
36	F	1,68	107,5 / 38,1	73,9 / 26.2

The patients had neuroglycopenic course with episodes of hypoglycemia in the late postoperative period, about six years after the procedure. The episodes of decreased level of consciousness and seizures initiated approximately 90-120 min after feeding, during which were documented capillary blood glucose of 35-45 mg/dl, which improved after parenteral infusion of glucose. During the etiological investigation it was found normality of serum C-peptide values, thus discarding the hypothesis factitious hyperinsulinemia. Glucose curve was performed from patients who showed hypoglycemia in the second hour after administration of glucose. Aiming to differentiate between insulinoma and NIPHS, was performed abdominal computed tomography with high resolution without evidence of the presence of focal pancreatic lesions. The realization of dietary and behavioral measures with nutritional and psychological support, as well as the addition of acarbose to clinical treatment, did not result in controlling symptoms, and the patients remained with multiple hospitalizations due to hypoglycemic events. Thus, it was proposed to carry out the surgical procedure to control the condition. Since there was refusal to employment pancreatic resection and the reversal of bariatric surgery, it was proposed treatment based on the pathophysiology of secondary NIPHS hyperinsulinemic hypoglycemia, jointly aiming at maintaining weight loss and resolution of preoperative comorbidities. The three patients had total symptom improvement and denied new episodes of hypoglycemia after revisional operation. The oral glucose tolerance tests confirmed the remission of the condition. Weight loss and resolution of comorbidities were held in all three cases, there was even extra weight loss in two of the patients. Table 2 shows comparison between BMI and laboratory examinations of patients before and after the surgical procedure.

**TABLE 2:** - Comparison between BMI and laboratory data before and after the revisional proposed surgery

**Patient 1**	**Pre-operative**	**Post-operative**
IMC	30.6	25.7
TOTG (0/ 30 / 60/ 90 /120 min)	84 / 200 / 202 / 144 / 45	80 / 136 / 142 / 112 / 89
Lipidogram (TG/LDL/ HDL)	94/ 112 / 47	93 / 103 / 50
HBA1C	4.8%	5.3%
Insuline	7,06	3.1
Peptide-C	1.4	1.1
PHmetry (DeMeester)	9.2	17,32
Follow-up	8 anos	29 meses

Patient 2	Pre-operative	Post-operative
IMC	30.1	26.5
TOTG (0/ 30 / 60/ 90 /120 min)	72 / 183 / 184 / 130 / 32	76 / 138 / 140 / 108 / 86
Lipidogram (TG/LDL/ HDL)	105 / 132 / 43	83 / 113 / 47
HBA1C	5.1%	5.7%
Insuline	8.2	6.0
Peptide-C	1.3	1.1
PHmetry (DeMeester)	4.1	12.21
Follow-up	9 anos	25 meses

Patient 3	Pre-operative	Post-operative
IMC	26.2	27.8
TOTG (0/ 30 / 60/ 90 /120 min)	78 / 191 / 172 / 91 / 42	83 / 139 / 148 / 98 / 92
Lipidogram (TG/LDL/ HDL)	132 / 96 / 39	112 / 98 /41
HBA1C	4.9%	5.1%
Insuline	12.1	3.2
Peptide-C	1.5	1.1
PHmetry (DeMeester)	2.02	9.83
Follow-up	11 anos	19 meses

BMI in kg/m²; TOTG=oral glucose tolerance test in mg/dL; lipidogram profile in mg/dl; insulin in microUI/ml; C-peptide in ng/ml; PH monitoring classified according to the DeMeester Score; follow-up preoperative considered since the completion of gastric bypass in Roux-en-Y

## DISCUSSION

Hypoglycemia with severe clinical symptoms occurs in about 1% of patients undergoing Roux-en-Y gastric bypass and it can happen in diabetic patients or not.

The main causes are the late dumping syndrome, factitious administration of insulin or oral hypoglycemic agents and NIPHS.

In the late dumping syndrome or reactive hypoglycemia may occur neuroglycopenic symptoms in response to high insulin release by eating foods with high glycemic index. Often there is an association with vasomotor manifestations, related to early dumping, which were most prevalent in symptoms. The resolution of the condition occurs with dietary readjustment and psychological support, without the use of pharmacological or surgical treatments.

In NIPHS the main symptoms are neuroglycopenic (changing level of consciousness and seizures), and generally occur 2-4 h after feeding. Hypoglycemic episodes should be documented and are usually below 45 mg/dl.

Fundamental part on the results on diabetes mellitus type 2 resolution - loss and weight maintenance after Roux-en-Y gastric bypass -, is related to the increased secretion of incretinic hormones, such as GIP, peptide-YY, oxyntomodulin and especially GLP 1.

Treatment of NIPHS is not defined. Initially, low-carbohydrate diet could benefit; however, this therapy is ineffective. As a second alternative proposal, apply some different medications as acarbose and octreotide. Drug treatment should be used in the dietary failure. In the absence of response to drug therapy, surgical therapy has been suggested.

In this case, there will be feasible and safe procedure, based on the trigger mechanism of the syndrome. The gastrectomy of the bypassed stomach aimed to be similar to the vertical gastrectomy, leaving a smaller gastric pouch and removing the orexigenic ghrelin stimulus. Staying there it could represent the major factor in weight regained.

The anastomosis of the feed loop to pre-pyloric antrum aims to reassume normal intestinal transit promoting change of the enteroinsular axis diminishing GIP and GLP-1 and hence hyperinsulinemia. 

It must be considered the possibility of regain weight in this operation. In this case, after the follow-up realized, the weight regain was not a rule, occurring even additional weight loss of 11.17 kg and 11.8 kg for two patients. This could be either by suppressing the production of ghrelin by the gastric fundus and by improvement in adherence to dietary and behavioral measures by patients. Postoperatively there was good progress with in-hospital control of episodes of hypoglycemia, persisting after discharge.

As a side effect, one of the patients complained of heartburn and acid regurgitation, being diagnosed gastroesophageal reflux disease with confirmation by esophageal pH monitoring with DeMeester score of 17.32. The symptoms were completely controlled with the use of proton pump inhibitor.

The current trend is that no more should be done subtotal pancreatectomy or almost complete, in view to morbidity and mortality of this operation, which often is not effective. Thus, one must make efforts to develop an effective surgical procedure for the treatment of hyperinsulinemic hypoglycemia after gastric bypass facing the real pathophysiology of the condition, trying to maintain the weight loss.

## CONCLUSION

The procedure to reestablish the food transit through the duodenum and proximal jejunum, keeping the restrictive component of gastric bypass in the treatment of hyperinsulinemic hypoglycemia, showed good initial results that can validate its use in other cases. 
